# Social Statistics Transformation: Understanding the population through the production of ethnicity statistics from administrative data

**DOI:** 10.23889/ijpds.v8i2.2248

**Published:** 2023-09-14

**Authors:** Alex Mylles, Charlie Collins

**Affiliations:** 1 Office for National Statistics, Newport, United Kingdom

## Objectives

The decennial censuses provide rich data on the ethnic composition of England and Wales. However, there is a need for more frequent statistics to allow us to better understand population change over time. Our ambition is producing annual population statistics by ethnic group down to local authority level and below.

## Method

Admin-based ethnicity statistics have been produced by linking ethnicity information from admin data (from schools, further education and the NHS) to an admin-based Statistical Population Dataset (our population base) to produce our best estimate of the population characteristics. This latest iteration of the research includes additional Welsh data and uses the most recent population base for 2021. A set of rules have been used to deal with multiple ethnicity records for a person, with focus groups conducted with the general public to gauge the public acceptability of our approach.

## Results

We will present the results from the latest version of the admin-based ethnicity statistics (to be published June 2023). The presentation explores the coverage achieved through linking together the multiple data sources and looks at how the admin-based estimates compare with the Census 2021 estimates. Earlier findings (published February 2023) indicate the admin-based estimates are getting closer to the Census figures, and that we have an ethnicity for approaching 9 out 10 people in the population. We hope to provide updated data showing the gap to Census decreasing and increased coverage. We will highlight some of the challenges in using administrative data sources to produce these statistics, in particular inconsistent coding of ethnicities across the admin datasets.

## Conclusion

We will show how this research has fed into the 2023 Census Consultation and discuss the planned next steps in the research to address our outstanding challenges. This includes outlining our plans for record-level comparisons with Census 2021 in order to assess the quality of the admin-based estimates.

